# ADHD and Neurodegenerative Disease Risk: A Critical Examination of the Evidence

**DOI:** 10.3389/fnagi.2021.826213

**Published:** 2022-01-25

**Authors:** Sara Becker, Manu J. Sharma, Brandy L. Callahan

**Affiliations:** ^1^Department of Psychology, University of Calgary, Calgary, AB, Canada; ^2^Hotchkiss Brain Institute, University of Calgary, Calgary, AB, Canada

**Keywords:** aging, Alzheimer disease, attention deficit disorder with hyperactivity, dementia, Parkinson disease, synucleinopathies, attention-deficit/hyperactivity disorder

## Abstract

In this review, we undertake a critical appraisal of eight published studies providing first evidence that a history of attention-deficit/hyperactivity disorder (ADHD) may increase risk for the later-life development of a neurodegenerative disease, in particular Lewy body diseases (LBD), by up to five-fold. Most of these studies have used data linked to health records in large population registers and include impressive sample sizes and adequate follow-up periods. We identify a number of methodological limitations as well, including potential diagnostic inaccuracies arising from the use of electronic health records, biases in the measurement of ADHD status and symptoms, and concerns surrounding the representativeness of ADHD and LBD cohorts. Consequently, previously reported risk associations may have been underestimated due to the high likelihood of potentially missed ADHD cases in groups used as “controls”, or alternatively previous estimates may be inflated due to the inclusion of confounding comorbidities or non-ADHD cases within “exposed” groups that may have better accounted for dementia risk. Prospective longitudinal studies involving well-characterized cases and controls are recommended to provide some reassurance about the validity of neurodegenerative risk estimates in ADHD.

## Introduction

Attention-deficit hyperactivity disorder (ADHD) is a psychiatric disorder beginning in childhood that is characterized by core symptoms of inattention, impulsivity, and hyperactivity (Biederman and Faraone, 2005; American Psychiatric Association, 2013; Faraone et al., 2015). Diagnostic criteria require symptoms to present in early childhood, before age 12, and cause impairment in daily activities in more than one setting (e.g., home, school, social environment, and/or interpersonal relationships; American Psychiatric Association, 2013). Although it is largely considered a childhood disorder, 40–60% of cases of ADHD persist into adulthood (Culpepper and Mattingly, 2010; Michielsen et al., 2012; Volkow and Swanson, 2013; Asherson et al., 2016), and the overall prevalence of adult ADHD ranges from 2 to 4% (Kieling and Rohde, 2012; Fayyad et al., 2017).

ADHD may persist into later life as well. Roughly 3% of adults over age 50 suffer from significant symptoms of attention-deficit/hyperactivity disorder (ADHD; Michielsen et al., 2012; Kooij et al., 2016), often presenting as executive dysfunction (e.g., absent-mindedness) and memory impairments (e.g., forgetfulness or difficulty learning new things; Rosler et al., 2010; Thorell et al., 2017; Callahan et al., 2021). These symptoms overlap with those of early neurodegenerative disease (Ivanchak et al., 2012; Pollack, 2012; Goodman et al., 2016; Callahan et al., 2017), and it is currently unclear whether ADHD is associated with an increased neurodegenerative risk, or if it is being misdiagnosed due to symptom overlap (Callahan et al., 2017). Clarifying this issue is crucial to reduce dementia misdiagnoses, and to guide treatment, which will differ depending on whether the disease course is assumed to be neurodegenerative or not.

Some data have suggested a link between ADHD and neurodegenerative disease, which appears to be specific to Lewy body diseases (LBD; Baumeister, 2021). Lewy body diseases refer inclusively to Parkinson’s disease, Parkinson’s disease dementia, and dementia with Lewy bodies, which are assumed to be different phenotypic expressions of underlying α-synuclein pathology (Gomperts, 2016). All three share clinical and neuropathological findings, including motor symptomatology (i.e., parkinsonism), progressive executive dysfunction, and non-motor symptoms such as autonomic dysfunction, sleep behavior disorders, hallucinations, and olfactory dysfunction (Aarsland, 2016; Sezgin et al., 2019). Motor abnormalities (Goulardins et al., 2017), executive dysfunction (Barkley, 1997), and disordered sleep (Konofal et al., 2010) are also present in ADHD, which has generated research interest in the relationship between these syndromes.

In the last 15 years, eight published studies have suggested that ADHD may be a risk factor for incident neurodegeneration (Walitza et al., 2007; Golimstok et al., 2011; Curtin et al., 2018; Fluegge and Fluegge, 2018; Tzeng et al., 2019; Fan et al., 2020; Du Rietz et al., 2021; Zhang et al., 2021; see Table 1). These studies have primarily used data from population registers with linked health information (Curtin et al., 2018; Du Rietz et al., 2021; Zhang et al., 2021), healthcare utilization databases (Fluegge and Fluegge, 2018), or health insurance databases (Tzeng et al., 2019), and have reported increased risk for neurodegeneration—particularly LBD—in individuals with antecedent ADHD. Two smaller retrospective case-control studies have similarly reported increased earlier-life ADHD symptoms in individuals with Parkinson’s disease relative to controls (Walitza et al., 2007; Golimstok et al., 2011). Hazard ratios in these studies range from 1.8 to 5.2 and odds ratios range from 1.5 to 5.1 (Table 1), suggesting strongly increased risk of later-life dementia in individuals with antecedent ADHD, even after controlling for potential confounds such as diabetes (Fluegge and Fluegge, 2018) or stimulant medication use (Curtin et al., 2018).

**Table 1:** Summary of eight studies investigating relationships between antecedent ADHD and later-life neurodegenerative diseases.

	Walitza et al. (2007)	Golimstok et al. (2011)	Curtin et al. (2018)	Fluegge and Fluegge (2018)	Tzeng et al. (2019)	Fan et al. (2020)	Du Rietz et al. (2021)	Zhang et al. (2021)
**Objective**	To quantify ADHD symptoms that preceded PD onset, accounting for exposure to psychostimulants.	To determine whether ADHD symptoms precede the onset of clinical DLB.	To investigate whether antecedent ADHD is linked to increased incidence of BG&C diseases.	To determine the role of antecedent ADHD in dementia risk.	To clarify the association between adults with ADHD and the risk of dementia.	To determine whether PD patients exhibit a greater propensity for prior ADHD than controls.	To investigate associations between ADHD and a wide range of physical health conditions across adulthood.	To evaluate cross-generation familial aggregation of ADHD and Alzheimer’s Disease/any dementia.
**Participants**	88 healthy controls 88 PD (43 early-onset)	149 healthy controls 109 DLB 251 AD	158,790 controls 31,769 ADHD	No sample sizes specified other than “162 state-year observations”	2,025 controls 675 ADHD	10,726 controls 10,726 PD	4,789,799 cases; 61,960 (1.29%) had lifetime prevalence of ADHD	2,132,929 index persons and their relatives (parents, grandparents, and aunt/uncles)
**Definition of ADHD**	WURS-k total score as the primary outcome of interest (also reported WURS-k ≥30 for clinical childhood diagnosis).	WURS ≥32 for retrospective diagnosis of childhood ADHD; DSM-IV criteria for diagnosis of adult ADHD.	ICD-9 codes 314.00 and 314.01 (ADD without and with hyperactivity), 314.1 (hyperkinesis with developmental delay), 314.2 (hyperkinetic conduct disorder), 314.8 and 314.9 (other or unspecified hyperkinetic manifestations)	ICD-9 code 314.01 (ADD with hyperactivity).	ICD-9 codes 314 (includes ADD with and without hyperactivity, hyperkinesis with developmental delay, hyperkinetic conduct disorder, other or unspecified hyperkinetic manifestations).	ICD-9-CM code 314.0 (ADD without and with hyperactivity).	ICD-9 codes 314 (includes ADD with and without hyperactivity, hyperkinesis with developmental delay, hyperkinetic conduct disorder, other or unspecified hyperkinetic manifestations) and ICD-10 codes F90 (includes disturbance of activity and attention, hyperkinetic conduct disorder, other or unspecified hyperkinetic disorder) or ADHD medication prescription.	ICD-9 codes 314 (includes ADD with and without hyperactivity, hyperkinesis with developmental delay, hyperkinetic conduct disorder, other or unspecified hyperkinetic manifestations) and F90 (includes disturbance of activity and attention, hyperkinetic conduct disorder, other or unspecified hyperkinetic disorder).
**Definition of later-life outcome**	Clinical diagnosis of PD.	Probable AD based on NINCDS/ ADRDA criteria (McKhann et al., 1984). DLB based on consensus criteria (McKeith et al., 1996).	ICD-9-CM codes 332.0 (PD), 332.1 (secondary parkinsonism), 333.0 (other degenerative basal ganglia diseases), 333.1 (essential and other specified forms of tremor).	ICD-9 codes 331.0 (AD) and 331.82 (LBD).	ICD-9-CM codes 290.0 (senile dementia), 290.10–290.13 (presenile dementia, uncomplicated or with delirium or delusional or depressive features), 290.20–290.21, 290.3 (senile dementia with delusional or depressive features or delirium), 290.4, 290.41–290.43 (vascular dementia, uncomplicated or with delirium or delusional or depressed mood), 290.8–290.9 (other or unspecified senile psychotic condition), and 331.0 (Alzheimer’s disease).	ICD-9-CM code 332.0 (PD) with at least 3 outpatient visits or hospital admissions and at least one PD medication.	ICD-8 codes 342.00 (PD), 342.08–342.09 (other defined or unspecified parkinsonism); ICD-9 codes 332.0 (PD), 332.1 (secondary parkinsonism), 333.0 (other degenerative diseases of the basal ganglia); ICD-10 codes G20 (PD), G21.2 (secondary parkinsonism due to other external agents), G21.3 (postencephalitic parkinsonism), G21.8-G21.9 (other defined or unspecified secondary parkinsonism), G23.1 (progressive supranuclear ophthalmoplegia), G23.2 (striatonigral degeneration), G23.8 (other specified degenerative diseases of basal ganglia), G23.9 (unspecified degenerative disease of basal ganglia), G25.9 (unspecified extrapyramidal and movement disorder).	AD: ICD-7 codes 304 (senile psychosis), 305 (presenile psychosis); ICD-8 code 290 (dementia senile and presenile); ICD-9 codes 290A (senile dementia), 290B (presenile dementia, onset < 65 years), 290X (dementia associated with aging, unspecified), 331A (presenile and senile degeneration of the Alzheimer’s type); ICD-10 codes F00 (dementia in AD), F03 (unspecified dementia), G30 (AD). Other dementias: ICD-7 code 306 (psychosis with arteriosclerosis of the brain); ICD-8 codes 293.0 (psychosis of central nervous system with cerebral arterioles), 293.1 (psychosis of central nervous system without cerebrovascular disease); ICD-9 codes 290E (multi-infarction dementia), 290W (other specified age-related dementia), 294B (dementia in somatic disease classified elsewhere), 331B (Pick’s syndrome), 331C (senile degeneration of the brain of unspecified type), 331X (cerebral degeneration, unspecified); ICD-10 codes F01 (vascular dementia), F02 (dementia in other diseases classified elsewhere), F05.1 (delirium with underlying dementia), G31.1 (senile degeneration of brain, not elsewhere classified), G31.8 (other specified degenerative diseases of nervous system).
**Covariates**	No participant was taking stimulant medication. No other covariates considered.	Patients matched to controls by age, sex and education. No participant was taking stimulant medication. No other covariates considered.	Adjusted for sex, age, race/ethnicity, psychotic conditions, tobacco use.	Adjusted for diabetes (clinical classification software diagnostic category 50) and obesity (not otherwise specified, code 278.00).	Adjusted for age, sex, comorbidities, geographical area, urbanization, income.	Adjusted for Charlson Comorbidity Index.	Adjusted for sex and birth year of both relatives (to account for different follow-up lengths).	Adjusted for birth year of index persons and of relatives, and sex of index persons and of relatives.
**Results**	On the WURS-k, group differences on the ‘Attention deficit/hyperactivity’ subscale (0.8 ± 0.8 in patients and 0.6 ± 0.6 in controls, *p* = 0.01). No differences in frequencies clinical childhood diagnosis of ADHD between patients (10.2%) and controls (6.8%, *p* =* 0*.418).	ADHD was significantly more frequent in DLB (47.8%) than in AD (15.2%) or controls (15.1%). OR_LBDvsControl_ = 5.1 [95% CI = 2.7–9.6].	Incident BG&C was significantly more frequent in ADHD (0.52%) than in controls (0.19%). aHR_BG&C_ = 2.4 [95% CI = 2.0–3.0]. Incident PD was significantly more frequent in ADHD (0.18%) than in controls (0.06%). aHR_PD_ = 2.6 [95% CI = 1.8–3.7]. When including only ADHD not taking stimulant medication: aHR_BG&C_ = 1.8 [95% CI = 1.4–2.3] and aHR_PD_ = 2.3 [95% CI = 1.5–3.5].	IRR_LBD_ = 1.16 [95% CI = 1.01–1.32] adjusted for diabetes. IRR_LBD_ = 1.06 [95% CI = 0.95–1.18, n.s.) adjusted for diabetes and obesity.	Incidence of dementia was higher in ADHD (5.48%) than in controls (4.0%). aHR_Dementia_ = 4.01 [95% CI = 2.53–6.36]. aHR_AD_ = 0.52 [95% CI = 0.06–4.53, n.s.]. aHR_VaD_ = 6.28 [95% CI = 2.71–25.85]. aHR_OtherDementia_ = 5.22 [95% CI = 3.13–8.72].	Prior diagnosis of ADHD was more frequent in PD (0.13%) than in controls (0.05%). Adjusted OR = 3.65 [95% CI = 2.26–10.50].	Individuals with ADHD had increased risk of all physical conditions except rheumatoid arthritis. OR_PD_ = 1.50 [95% CI = 1.08–2.09]. OR_Dementia_ = 2.44 [95% CI = 1.86–3.19].	Higher risk of AD and dementia in family members of index persons with ADHD compared to family members of index persons without ADHD. Parents: aHR_AD_ = 1.55 [95% CI = 1.26–1.89]). aHR_AnyDementia_ = 1.34 [95% CI = 1.11–1.63]. Grandparents: aHR_AD_ = 1.11 [95% CI = 1.08–1.13]. aHR_AnyDementia_ = 1.10 [95% CI = 1.08–1.12].

*Notes. –: not reported/not applicable. AD, Alzheimer’s Disease/Dementia; ADD, Attention Deficit Disorder; ADHD, Attention-Deficit Hyperactivity Disorder; aHR, adjusted Hazards ratio; BG&C, Basal Ganglia and Cerebellar Disorders; CI, Confidence Interval; CM, Clinical Modification; DSM-IV, Diagnostic and Statistical Manual of Mental Disorders Fourth Edition; ICD, International Classification of Diseases; IRR, incidence rate ratio; LBD, Lewy Body Diseases; OR, Odds Ratio; PD, Parkinson’s Disease; WURS-k, Wender Utah Rating Scale—German short version*.

A recent review (Baumeister, 2021) summarized findings from five of these eight studies, and thoroughly described alterations in dopaminergic, cognitive, and neural functioning that are common to both ADHD and Parkinson’s disease in an attempt to understand the etiology and pathogenesis of both disorders. Here, we extend this discussion to address three new studies published in 2020 and 2021, and take a more critical perspective in appraising this literature. We argue that the evidence presented in these eight studies deserves very careful consideration, because its corollary is that ADHD may represent a preclinical or risk stage of neurodegeneration during which important decision-making and early intervention may be possible.

## Critical Appraisal of Methodological Approaches

### Electronic Health Records

The use of health administrative data enables population-level analyses, improves generalizability of results, and minimizes referral bias (Benchimol et al., 2011). The systematic collection of data over time is particularly beneficial for studying both neurodevelopmental and neurodegenerative disorders. However, there are limitations in using such data, including misclassifications and coding or data entry errors, as well as changes in both diagnostic criteria and coding systems over time (Mazzali et al., 2016).

In four studies (Curtin et al., 2018; Fluegge and Fluegge, 2018; Tzeng et al., 2019; Du Rietz et al., 2021), ADHD participants were identified *via* health record diagnostic codes from the International Classification of Diseases (ICD), and the authors evaluated the risk of subsequent basal ganglia diseases including LBD (Curtin et al., 2018; Du Rietz et al., 2021) or Lewy body or Alzheimer’s dementias (Fluegge and Fluegge, 2018; Tzeng et al., 2019; Du Rietz et al., 2021) associated with these codes. Another study (Zhang et al., 2021) investigated the relationship between familial aggregation of ADHD and dementia using both ICD-9 and ICD-10 codes. Not all studies, however, included consistent or intuitive operationalizations of ADHD (Table 1). For instance, two (Tzeng et al., 2019; Zhang et al., 2021) considered inattentive, hyperactive, and combined ADHD presentations (codes 314.00-01), while another (Fluegge and Fluegge, 2018) only considered hyperactivity presentations (314.01). Thus, ADHD cases presenting without hyperactivity—which represent between 20% (Salvi et al., 2019) and 40% (Gibbins et al., 2010) of adult cases—were not captured in the Fluegge and Fluegge (2018) study, suggesting the sample was an incomplete representation of ADHD. In contrast, two other studies (Curtin et al., 2018; Du Rietz et al., 2021) included hyperkinetic disorders within their ADHD designation (codes 314.0–314.2, 314.8, 314.9), which is problematic because the criteria for these categories are not well defined, and misdiagnoses are common [e.g., hyperkinetic movement disorders may be mistaken for tics or Tourette’s syndrome (Kompliti and Goetz, 1998)]. Hyperkinetic disorders are also characterized by excessive involuntary movements resulting from basal ganglia damage (Wichmann and DeLong, 2010), so their consideration as a risk factor for basal ganglia diseases such as LBD is somewhat tautological.

There is also considerable variability in the disorders encompassed by the diagnostic codes used to define LBD (Table 1). For example, two studies considered “secondary parkinsonism” (Curtin et al., 2018; Du Rietz et al., 2021) among the outcomes of interest, however secondary parkinsonism is not necessarily neurodegenerative (Shin and Chung, 2012) and may have potentially confounded results. The multiple aetiologies present within the outcome samples result in a very heterogenous group of diagnoses that may not be particularly attributed to LBD, and precludes robust conclusions about the specific association between ADHD and neurodegenerative LBD.

There is also considerable worldwide variability in the use of the ICD versions, with some countries implementing “clinical modifications” that modify ICD codes according to country-specific needs, e.g., ICD-9-CM and ICD-10-CM in the US, or ICD-10-CA in Canada (Otero Varela et al., 2021). These modifications can either include more detailed coding to characterize a patient’s state (generating more individual codes than the original version) or be used to simplify the index terms of the ICD. Overall, this leads to variability in the number of codes and categories, as well as definitions in each modified ICD (Jetté et al., 2010), which compromises comparability of data between countries.

The extent to which ICD codes actually correspond to the presence of a disease or disorder has been examined in multiple studies of ADHD (e.g., O’Malley et al., 2005; Daley et al., 2017) and neurogenerative diseases (Wermuth et al., 2012; Muzerengi et al., 2017; Wilkinson et al., 2018; Harding et al., 2019), and misdiagnosis rates as high as 80% have been reported (O’Malley et al., 2005). A systematic review found that healthcare data had low sensitivity (<50%) for identifying all types of dementia (Wilkinson et al., 2018). The diagnosis of AD or LBD relies heavily on complex clinical criteria (McKhann et al., 2011; McKeith et al., 2017) which makes them more difficult to code than conditions where specific diagnostic tests can confirm the diagnosis [e.g., diabetes (St. Germaine-Smith et al., 2012)]. An examination of ICD codes in the Danish National Hospital Register (Wermuth et al., 2012) found that 18% of patients coded as having idiopathic Parkinson’s disease did not meet consensus criteria upon careful review of clinical features. Especially in Parkinson’s disease, misdiagnoses are common (Feldman et al., 2012; Harding et al., 2019), calling into question the use of different clinical standards across different health registries and individual health centers. A previous study examining the accuracy of ICD-9-CM codes in identifying cases of parkinsonism found that using medications listed in pharmacy data was more accurate than using medical records (Swarztrauber et al., 2005), which was also corroborated by a Swedish study (Hjerpe et al., 2010).

Error rates in ICD coding for ADHD may also be high, in part because clinicians seldom actually establish that all ADHD diagnostic criteria are present, even when an ICD code of ADHD is recorded consistently across visits (Daley et al., 2017). At most, only about half of clinicians explicitly quantify ADHD symptoms, and fewer than 15% formally document whether symptoms fall above the clinical threshold (Daley et al., 2017). Further errors can be introduced because ICD codes are generally not assigned by clinicians themselves, but by coders based on clinicians’ notes (O’Malley et al., 2005), and therefore depend on the clarity and consistency of the terminology used in the notes. As such, ICD codes do not always reflect a clear and systematic ascertainment of symptom severity, functional impairment, childhood onset, or ruling out other possible aetiologies. This is rather problematic in the context of examining relationships between ADHD and neurodegeneration, because ADHD-like experiences (inattention, distractibility, impulsivity, and emotional dysregulation) can be neuropsychiatric indicators of neurodegenerative disease onset (Ismail et al., 2018; Bateman et al., 2020), and establishing whether they are due to ADHD or to neurodegeneration is key.

The selection of controls in these studies does not include systematic assessment to rule out ADHD. This is problematic because, especially in adults, ADHD is underdiagnosed (Newcorn et al., 2007; Ginsberg et al., 2014). In one article (Tzeng et al., 2019), ADHD prevalence was 0.07%, whereas worldwide adult prevalence is consistently closer to 3% (Polanczyk et al., 2007; Kooij et al., 2016), suggesting that up to 2.9% of the “non-ADHD” group may in fact have had ADHD, for overall rates to be on par with expected prevalence. A recent study (Chen et al., 2019) using the same Taiwanese National Health Insurance registry identified 275,980 ADHD participants over 12 years (23,000 new cases annually), significantly more than the 675 ADHD cases identified within the 1-year enrolment period in Tzeng et al. (2019), suggesting many missed cases in Tzeng and colleagues’ investigation.

Lastly, it should be noted that two studies in this review used data from either inpatient hospitalization visits (Fluegge and Fluegge, 2018) or a minimum of three outpatient visits (Tzeng et al., 2019), which likely biased the samples towards severe ADHD or patients with chronic illnesses or health comorbidities resulting in increased doctor’s visits. Indeed, three annual outpatient visits is uncommon for adults with ADHD; the mean is closer to one annual visit (Garcia-Argibay et al., 2021). Additional comorbidities leading to multiple hospital visits may account for dementia risk in these studies (Vassilaki et al., 2015), particularly in older adults for whom comorbidities are increased (Chen et al., 2018). Moreover, evaluating ADHD as a predictor of *hospitalization* for LBD or AD is not equivalent to evaluating it as a predictor of the *development* of these conditions, especially as ADHD on its own is recognized to be associated with an increased risk of hospitalization (Chien et al., 2017; Lindemann et al., 2017). As such, the methodological approaches used in these studies preclude the authors’ inference that there exists an “association between antecedent ADHD and dementia risk” (Fluegge and Fluegge, 2018).

### Definitions of ADHD

In two case-control studies of interest (Walitza et al., 2007; Golimstok et al., 2011), the researchers did not actually diagnose ADHD but rather ascertained symptom frequencies and used cut-off scores to classify participants into “ADHD” and “non-ADHD” groups. Symptom rating scales provide data that must be taken as only one component of a broad clinical assessment (Ramsay, 2017) including a comprehensive clinical history and corroborating reports (Sibley et al., 2012; Sibley, 2021). Further, traits of inattention, hyperactivity and impulsivity are endorsed by 60% of adults (Das et al., 2012), and their use cannot provide any conclusions about whether clinical ADHD is associated with neurodegenerative disease outcomes. It is also possible that some adults may overreport normal cognitive or behavioral fluctuations as symptoms of ADHD, or experience distractibility or disorganization in the absence of clinically significant impairment, both of which preclude a clinical diagnosis of ADHD (Sibley, 2021).

In quantifying childhood symptoms, neither study considered potential confounding factors that may have accounted for symptomatology. It is crucial to ascertain that inattention/hyperactivity in childhood is truly attributable to ADHD and not to other factors such as traumatic brain injury (Lee et al., 2013) or Asperger syndrome (Tani et al., 2006), for example. Ruling out alternative explanations for symptoms is a key step in ensuring diagnostic accuracy when assessing ADHD in adults (Sibley, 2021), and failure to do so often results in an overestimation of symptoms attributable to ADHD (Sibley et al., 2017). Inquiring about these antecedents is also critical to determine whether they can account for any significant variance in neurodegenerative disease risk. Brain injury, hypertension, physical inactivity, cardiovascular risk factors, and excessive alcohol and tobacco consumption have all been linked to dementia risk (Bergland et al., 2017; Livingston et al., 2020) and many have been shown to be increased in ADHD as well (Callahan et al., 2017). As such, they should be considered potential confounds and should be controlled for in study designs or statistical models.

### Representativeness of Patient Samples

In a smaller case control study (Golimstok et al., 2011), participants were identified as positive cases of ADHD if they demonstrated both current and childhood symptoms. As a result, cases of remitted ADHD—generally thought to represent at least 50–60% of ADHD cases (Sibley et al., 2017) —were unaccounted for. It is unclear if or how the dementia and control groups differed in their prevalence of remitted ADHD, but this has important implications for understanding relationships between ADHD in early life and later neurodegenerative disease risk. Notably, prevalence of ADHD in this study was roughly five times higher in the control group (15.1%) relative to expected frequencies of ADHD in the general adult or older adult population (~3%; Michielsen et al., 2012; Kooij et al., 2016), strongly implying faulty recall and/or an unrepresentative sample.

In examining neurodegenerative disease outcomes, some studies included early-onset disease cases (Walitza et al., 2007; Curtin et al., 2018). This may introduce bias, because specific genetic mutations account for early-onset LBD (Escott-Price et al., 2015; Ylönen et al., 2017) and these patients are genotypically and phenotypically different from patients with later onset (Schrag and Schott, 2006; Ferguson et al., 2016; Post et al., 2020). In one study (Walitza et al., 2007), the early-onset group comprised 48.9% of all patients with Parkinson’s disease, yet the population prevalence of early-onset cases is approximately 5–10% of all Parkinson’s disease cases (Wickremaratchi et al., 2009; Mehanna et al., 2014), suggesting the sample may not be representative of, or generalizable to, Parkinson’s disease as a whole. Notably, the sex distribution in Curtin and colleagues’ (Curtin et al., 2018) Parkinson’s disease sample (44% female) is not typical of this disorder, in which males outnumber females 1.5–2:1 (Haaxma et al., 2007), also calling into question the representativeness of their sample.

No studies accounted for attrition, despite acknowledgements that at least some participants withdrew from initial eligible samples (Tzeng et al., 2019) and despite the fact that attrition rates are typically quite high in longitudinal studies of ADHD (e.g., Hartsough et al., 1996). Only one of the studies (Golimstok et al., 2011) considered education in their analyses, despite the fact that adults with ADHD typically have lower educational attainment (Biederman et al., 2008) which may account for important variance in dementia risk (Sharp and Gatz, 2011). Furthermore, previous research has shown that males have a two- to four-times higher risk of developing a neurodevelopmental disorder such as ADHD than females (May et al., 2019), and males have a higher risk of Parkinson’s disease than females (Haaxma et al., 2007), while there is a slightly higher risk for females of developing dementia with Lewy bodies (Mouton et al., 2018). Despite these sex differences, only three of the studies considered sex as a covariate in their statistical models (Curtin et al., 2018; Tzeng et al., 2019; Du Rietz et al., 2021) and one matched groups on sex (Golimstok et al., 2011). Lastly, all articles except two (Tzeng et al., 2019; Fan et al., 2020) disregarded obvious risk factors for developing dementia, such as traumatic brain injury, hypertension, physical inactivity, cardiovascular risk factors, excessive alcohol consumption, and smoking (Bergland et al., 2017; Livingston et al., 2020). Many of these risk factors are increased in ADHD, including traumatic brain injury (Liou et al., 2018), hypertension (Fuemmeler et al., 2011), cardiovascular risk factors (Cortese et al., 2013; Irmisch et al., 2013; Chen et al., 2018; Du Rietz et al., 2021), and smoking (Galéra et al., 2017), and these factors may be accounting for a substantial portion of the putative association between ADHD and later-life neurodegenerative disease (as in Bendayan et al., 2020, for example).

### Recall Biases

In two studies (Walitza et al., 2007; Golimstok et al., 2011), participants with mild dementia and healthy controls were asked to self-rate ADHD symptoms during early childhood, to determine whether antecedent ADHD was more frequent in the mild dementia group. In one study (Golimstok et al., 2011), corroborating evidence may have been supplemented by an informant who knew the participant for at least 10 years but not necessarily since childhood. In our view (Sharma et al., 2021), retrospective self-report in older adults—requiring recall from >40 years prior—severely compromises the validity of the data being collected, particularly in samples composed of individuals with cognitive impairment. Even young adults are poor historians of their childhood ADHD symptoms: nearly 40% of 22-year-old adults who do not recall their childhood symptoms actually did have ADHD as children, and roughly 60% remember symptoms that, in fact, were not present (Breda et al., 2020). A possible recall bias is supported by one study’s data (Walitza et al., 2007): healthy control participants (mean age 57.2 years) reported significantly fewer ADHD symptoms relative to the younger healthy normative sample (mean age 29.8 years: Retz-Junginger et al., 2002), whereas it has been established that population samples of younger and older adults should endorse similar rates of ADHD symptoms (Callahan and Plamondon, 2019). Self-report is also less sensitive than informant-report for detecting current and past ADHD symptoms (Zucker et al., 2002; Sibley et al., 2012). Additionally, it is unknown whether participants in these studies were blinded to the study’s aims; being “cued” about possible links between antecedent ADHD and their current condition (LBD) may have inflated their retrospective recall of childhood symptoms.

### Balance of Strengths and Weaknesses

We conducted a formal evaluation of the quality of the eight studies in this review using the Newcastle-Ottawa Scale (Wells et al., 2011), and found many strengths across each (Table 2). All case-control studies had clear criteria for the selection of controls, and two ensured that participants were age- and sex-matched (Golimstok et al., 2011; Fan et al., 2020). Consensus criteria were used in two of the three case-control studies to ascertain neurodegenerative disease outcomes (Walitza et al., 2007; Golimstok et al., 2011). Among cohort studies, strengths included participants drawn from large population cohorts and usually matched on age and sex (or analyses were adjusted for these factors). Follow-up length was at least 10 years in four studies (Curtin et al., 2018; Fluegge and Fluegge, 2018; Tzeng et al., 2019; Du Rietz et al., 2021).

**Table 2:** Evaluation of the quality of the eight studies in this review using the Newcastle-Ottawa Scale.

	Case-control studies								
	Is the case definition (ND outcome) adequate?	Representativeness of the cases	Selection of controls	Definition of controls	Comparability of groups	Ascertainment of exposure	Same method of ascertainment for cases and controls?	Non-response rate	Overall quality
Walitza et al. (2007)	Known medical diagnosis 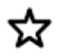	No; sample was enriched for early-onset PD	Community controls 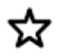	No history of PD 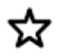	Groups not matched, and no adjustment for confounds	Symptom count in childhood based on retrospective self-report	Yes 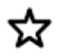	Same in both groups (0%) 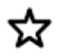	5/9
Fan et al. (2020)	ICD 9-CM code of PD with ≥3 outpatient visits or hospital admissions and receiving PD medication	Yes 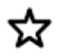	Same sample as cases, but unclear if controls constitute a hospitalized sample	Figure 1 indicates controls were ‘subjects without PD’ 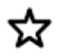	Groups matched on sex, age, and index date; analyses used Charlson Comorbidity Index 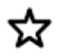 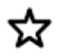	ICD-9-CM code of 314.0 (ADD with and without hyperactivity)	Yes 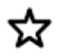	Same in both groups (0%) 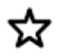	6/9
Golimstok et al. (2011)	Probable AD based on NINCDS/ADRDA criteria (Lindemann et al., 2017). DLB based on consensus criteria (Chien et al., 2017); diagnoses based on several sources 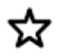	Yes 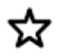	Same sample as cases, but unclear if controls constitute a hospitalized sample	No history of ND 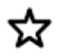	Groups matched on sex, age, geographic area, and education 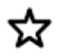 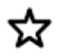	DSM-IV criteria ascertained by clinician blind to case/control status 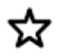 Retrospective self-report	Yes 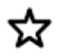	Same in both groups (0%) 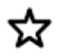	8/9
	**Cohort studies**								
	**Representativeness of the exposed cohort**	**Selection of the non-exposed cohort**	**Ascertainment of exposure^a^**	**Demonstration that outcome of interest was not present at start of study**	**Comparability of cohorts on the basis of the design or analysis**	**Assessment of outcome**	**Was follow-up long enough for outcomes to occur**	**Adequacy of follow up of cohorts**	
Curtin et al. (2018)	Included several non-ADHD hyperkinetic syndromes	Drawn from the same population as the exposed cohort 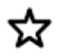	ICD 9-CM codes linked to Utah Population Database 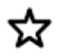	Patients were excluded if BG&C disorders were present prior to an index ADHD diagnosis or before age 21 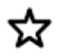	Matched on sex and birth year; analyses controlled for race, ethnicity, psychotic conditions and tobacco use 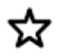 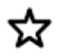	ICD 9-CM codes linked to Utah Population Database 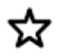	1996–2016 (median follow-up was 21 years) 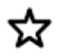	2.5% cases lost to follow-up vs. <1% controls; statistical models included a competing risk of death 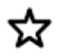	8/9
Fluegge and Fluegge (2018)	Only considered ADD with hyperactivity (not inattentive presentation); only considered hospitalization for ADHD	Drawn from the same population as the exposed cohort 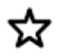	ICD 9-CM codes linked to the Healthcare Cost and Utilization Project 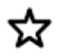	No	Not stated whether cohorts were comparable; analyses adjusted for age, diabetes and obesity 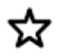 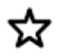	ICD 9-CM codes linked to the Healthcare Cost and Utilization Project 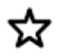	Ten-year lagged measure 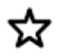	Data were drawn from the HCUP, which includes *all* discharge records since 1988 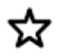	7/9
Tzeng et al. (2019)	Included several non-ADHD hyperkinetic syndromes; exposed cohort restricted to inpatients, or those with ≥3 outpatient visits within 1 year	Drawn from the same population as the exposed cohort 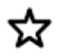	ICD 9-CM codes linked to the National Health Insurance Program 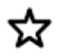	Participants excluded if dementia was present before tracking began or before an ADHD diagnosis 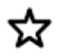	Matched on sex, age, geographic area and urbanization of residence, comorbidities, and income 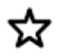 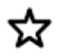	ICD 9-CM codes linked to the National Health Insurance Program 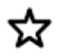	2000–2010 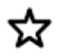	No information provided	7/9
Du Rietz et al. (2021)	Included several non-ADHD hyperkinetic syndromes or individuals prescribed ADHD medication	Siblings, half-siblings, and family members, drawn from the same population as the exposed cohort 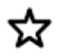	ICD-9 and ICD-10 codes linked to the National Patient Register 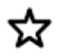	No, exposures and outcomes were treated as lifetime presence or absence (no consideration of onset timing)	Stratified by sex, and birth year of relatives to adjust for follow-up lengths 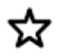 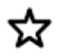	ICD-9 and ICD-10 codes linked to the National Patient Register 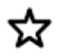	All participants followed from birth until 2013, range 18–81 years (mean = 47 years) 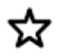	Data were drawn from the Total Population Register (i.e., includes *all* persons living in Sweden) 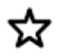	7/9
Zhang et al. (2021)	Included several non-ADHD hyperkinetic syndromes or individuals prescribed ADHD medication	Drawn from the same population as the exposed cohort 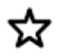	ICD-9 and ICD-10 codes linked to the National Patient Register 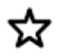	Not applicable, as the aim was to evaluate dementia in biological relatives, not individuals with ADHD themselves.	Analyses adjusted for index persons’ and relatives’ birth year and sex 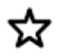 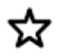	ICD-7, ICD-8, ICD-9, and ICD-10 codes linked to the National Patient Register 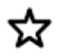	All relatives followed until dementia onset, death, migration, or end of study (median 8–25 years) 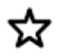	Data were drawn from the Total Population Register (i.e., includes *all* persons living in Sweden) 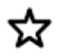	7/8

*Notes. ^a^The Newcastle-Ottawa Scale considers record linkage using ICD codes to be sufficient to confirm certain outcomes of interest, despite the limitations of this methodology to identify neurodegenerative diseases described previously (Wilkinson et al., 2018; Harding et al., 2019) and summarized in the manuscript text. AD, Alzheimer’s Disease/Dementia; ADD, Attention Deficit Disorder; ADHD, Attention-Deficit Hyperactivity Disorder; BG&C, Basal Ganglia and Cerebellar Disorders; CM, Clinical Modification; DSM-IV, Diagnostic and Statistical Manual of Mental Disorders Fourth Edition; HCUP, Healthcare Cost and Utilization Project; ICD, International Classification of Diseases; LBD, Lewy Body Diseases; PD, Parkinson’s Disease. 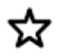
Indicates a high-quality choice, with a maximum of one star in all categories except “Comparability” which has a two-star maximum*.

Despite these strengths, two major components required to draw strong conclusions from these studies—ascertainment of exposure (ADHD) and representativeness of the exposed cohort—were flawed across almost all studies: including non-ADHD hyperkinetic syndromes or restricting the sample to those with numerous outpatient visits or hospitalizations results in samples that do not likely represent most ADHD cases, and non-exposed cases were also not systematically assessed to rule out ADHD. Likely, these methodological issues result in a conservative estimate of true effects; that is, without the “noise” generated by the inclusion of non-ADHD cases in the exposed group and potentially missed ADHD in the non-exposed group, associations between exposure and outcome may be even stronger. A second possibility is that the inclusion of non-ADHD cases in the exposed group is driving observed effects. As previously noted, many non-ADHD hyperkinetic disorders—which were included in the exposed cohort in four of the five cohort studies (Curtin et al., 2018; Tzeng et al., 2019; Du Rietz et al., 2021; Zhang et al., 2021)—are caused by damage to the basal ganglia (Wichmann and DeLong, 2010) and may conceivably predispose one to further later-life basal ganglia dysfunction (a hallmark of LBD, e.g., McKeith et al., 2017). To tease apart these issues will require careful and systematic assessment of ADHD across all participants within future studies, and an examination of neurodegenerative disease risk in ADHD and non-ADHD hyperkinetic syndromes separately.

## Conclusions

These studies present first evidence of a link between ADHD and risk of dementia, specific to LBD. Strengths include well-powered analyses and extensive follow-up periods (>10 years) in most studies. However, six (Curtin et al., 2018; Fluegge and Fluegge, 2018; Tzeng et al., 2019; Fan et al., 2020; Du Rietz et al., 2021; Zhang et al., 2021) rely on ICD diagnostic codes extracted from electronic health records, and there are limitations around the validity and accuracy of these codes, particularly for a disorder that is notoriously difficult to detect in adults (Newcorn et al., 2007; Ginsberg et al., 2014). The remaining two (Walitza et al., 2007; Golimstok et al., 2011) are limited by potentially biased and inaccurate self-reported retrospective childhood ADHD symptoms in small samples of cognitively impaired participants.

These studies provide tentative support for ADHD as a potential risk factor for later development of a neurodegenerative disease. Due to the methodological limitations and biases we have identified, we argue that the true association between ADHD and neurodegeneration is not yet identifiable. The sources of biases identified here should be considered in future studies to ascertain the true relative risk of neurodegeneration in patients with ADHD. Accurate and systematic diagnoses of ADHD and neurodegeneration are needed.

## Author Contributions

BC conceptualized the work. SB, MS, and BC made substantial contributions to the critical analysis and interpretation of data. SB, MS, and BC wrote initial drafts of the manuscript. SB and BC wrote the final draft. All authors revised it critically for important intellectual content, approved the version to be published, and agree to be accountable for all aspects of the work.

## Conflict of Interest

The authors declare that the research was conducted in the absence of any commercial or financial relationships that could be construed as a potential conflict of interest.

## Publisher’s Note

All claims expressed in this article are solely those of the authors and do not necessarily represent those of their affiliated organizations, or those of the publisher, the editors and the reviewers. Any product that may be evaluated in this article, or claim that may be made by its manufacturer, is not guaranteed or endorsed by the publisher.

## References

[B1] Aarsland D. (2016). Cognitive impairment in Parkinson’s disease and dementia with Lewy bodies. Parkinsonism Relat. Disord. 22, S144–S148. 10.1016/j.parkreldis.2015.09.034 26411499

[B2] American Psychiatric Association (2013). Diagnostic and Statistical Manual of Mental Disorders, 5th Edn. Arlington, VA: American Psychiatric Publishing.

[B3] Asherson P. Buitelaar J. Faraone S. V. Rohde L. A. (2016). Adult attention-deficit hyperactivity disorder: key conceptual issues. Lancet Psychiatry 3, 568–578. 10.1016/S2215-0366(16)30032-3 27183901

[B4] Barkley R. (1997). Behavioral inhibition, sustained attention and executive functions: constructing a unifying theory of ADHD. Psychol. Bull. 121, 65–94. 10.1037/0033-2909.121.1.65 9000892

[B5] Bateman D. R. Gill S. Hu S. Foster E. D. Ruthirakuhan M. T. Sellek A. F.. (2020). Agitation and impulsivity in mid and late life as possible risk markers for incident dementia. Alzheimer’s Dement. (N Y) 6:e12016. 10.1002/trc2.12016 32995467 PMC7507499

[B6] Baumeister A. A. (2021). Is attention-deficit/hyperactivity disorder a risk syndrome for Parkinson’s disease. Harv. Rev. Psychiatry 29, 142–158. 10.1097/HRP.0000000000000283 33560690

[B7] Benchimol E. I. Manuel D. G. To T. Griffiths A. M. Rabeneck L. Guttmann A. (2011). Development and use of reporting guidelines for assessing the quality of validation studies of health administrative data. J. Clin. Epidemiol. 64, 821–829. 10.1016/j.jclinepi.2010.10.006 21194889

[B8] Bendayan R. Mascio A. Stewart R. Roberts A. Dobson R. J. (2020). Cognitive trajectories in comorbid dementia with schizophrenia or bipolar disorder: the south london and maudsley nhs foundation trust biomedical research centre (SLaM BRC) case register. Am. J. Geriatr. Psychiatry 29, 604–616. 10.1016/j.jagp.2020.10.018 33250337 PMC8169045

[B9] Bergland A. K. Dalen I. Larsen A. I. Aarsland D. Soennesyn H. (2017). Effect of vascular risk factors on the progression of mild Alzheimer’s disease and lewy body dementia. J. Alzheimer’s Dis. 56, 575–584. 10.3233/JAD-160847 28035932

[B10] Biederman J. Faraone S. V. (2005). Attention-deficit hyperactivity disorder. Lancet 366, 237–248. 10.1016/S0140-6736(05)66915-2 16023516

[B11] Biederman J. Petty C. R. Fried R. Kaiser R. Dolan C. R. Schoenfeld S.. (2008). Educational and occupational underattainment in adults with attention-deficit/hyperactivity disorder: a controlled study. J. Clin. Psychiatry 69, 1217–1222. 10.4088/jcp.v69n0803 18681752

[B12] Breda V. Rohde L. A. Menezes A. M. B. Anselmi L. Caye A. Rovaris D. L.. (2020). Revisiting ADHD age-of-onset in adults: to what extent should we rely on the recall of childhood symptoms. Psychol. Med. 50, 857–866. 10.1017/S003329171900076X 30968792

[B13] Callahan B. L. Bierstone D. Stuss D. T. Black S. E. (2017). Adult ADHD: risk factor for dementia or phenotypic mimic. Front. Aging Neurosci. 9, 1–15. 10.3389/fnagi.2017.00260 28824421 PMC5540971

[B14] Callahan B. L. Plamondon A. (2019). Examining the validity of the ADHD concept in adults and older adults. CNS Spectr. 24, 518–525. 10.1017/S1092852918001190 30295232

[B15] Callahan B. L. Ramakrishnan N. Shammi P. Bierstone D. Taylor R. Ozzoude M.. (2021). Cognitive and neuroimaging profiles of older adults with attention deficit/hyperactivity disorder presenting to a memory clinic. J. Atten. Disord. 10.1177/10870547211060546. [Online ahead of print]. 34784815 PMC9066671

[B16] Chen V.C.-H. Chan H.-L. Wu S.-I. Lee M. Lu M.-L. Liang H.-Y.. (2019). Attention-deficit/hyperactivity disorder and mortality risk in Taiwan. JAMA Netw. Open. 2:e198714. 10.1001/jamanetworkopen.2019.8714 31390039 PMC6686778

[B18] Chen Q. Hartman C. A. Haavik J. Harro J. Klungsøyr K. Hegvik T.-A.. (2018). Common psychiatric and metabolic comorbidity of adult attention-deficit/hyperactivity disorder: a population-based cross-sectional study. PLoS One 13:e0204516. 10.1371/journal.pone.0204516 30256837 PMC6157884

[B17] Chen M.-H. Pan T.-L. Hsu J.-W. Huang K.-L. Su T.-P. Li C.-T.. (2018). Risk of type 2 diabetes in adolescents and young adults with attention-deficit/hyperactivity disorder. J. Clin. Psychiatry 79:17m11607. 10.4088/JCP.17m11607 29727071

[B19] Chien W.-C. Chung C.-H. Lin F.-H. Yeh C.-B. Huang S.-Y. Lu R.-B.. (2017). The risk of injury in adults with attention-deficit hyperactivity disorder: a nationwide, matched-cohort, population-based study in Taiwan. Res. Dev. Disabil. 65, 57–73. 10.1016/j.ridd.2017.04.011 28458048

[B21] Cortese S. Faraone S. V. Bernardi S. Wang S. Blanco C. (2013). Adult attention-deficit hyperactivity disorder and obesity: epidemiological study. Br. J. Psychiatry 203, 24–34. 10.1192/bjp.bp.112.123299 23661765 PMC3696877

[B22] Culpepper L. Mattingly G. (2010). Challenges in identifying and managing attention-deficit/hyperactivity disorder in adults in the primary care setting: a review of the literature. Prim. Care Companion J. Clin. Psychiatry 12, e1–e17. 10.4088/PCC.10r00951pur 21494335 PMC3067998

[B23] Curtin K. Fleckenstein A. E. Keeshin B. R. Yurgelun-Todd D. A. Renshaw P. F. Smith K. R.. (2018). Increased risk of diseases of the basal ganglia and cerebellum in patients with a history of attention-deficit/hyperactivity disorder. Neuropsychopharmacology 43, 2548–2555. 10.1038/s41386-018-0207-5 30209407 PMC6224615

[B24] Daley M. F. Newton D. A. DeBar L. Newcomer S. R. Pieper L. Boscarino J. A.. (2017). Accuracy of electronic health record-derived data for the identification of incident ADHD. J. Atten. Disord. 21, 416–425. 10.1177/1087054713520616 24510475

[B25] Das D. Cherbuin N. Butterworth P. Anstey K. Easteal S. (2012). A population-based study of attention deficit/hyperactivity disorder symptoms and associated impairment in middle-aged adults. PLoS One 7, 1–9. 10.1371/journal.pone.0031500 22347487 PMC3275565

[B26] Du Rietz E. Brikell I. Butwicka A. Leone M. Chang Z. Cortese S.. (2021). Mapping phenotypic and aetiological associations between ADHD and physical conditions in adulthood in Sweden: a genetically informed register study. Lancet. Psychiatry 8, 774–783. 10.1016/S2215-0366(21)00171-1 34242595 PMC8376653

[B27] Escott-Price, V. for International Parkinson’s Disease Genomics Consortium Nalls M. A. Morris H. R. Lubbe S. Brice A. Gasser T.. (2015). Polygenic risk of Parkinson disease is correlated with disease age at onset. Ann. Neurol. 77, 582–591. 10.1002/ana.24335 25773351 PMC4737223

[B28] Fan H.-C. Chang Y.-K. Tsai J.-D. Chiang K.-L. Shih J.-H. Yeh K.-Y.. (2020). The association between Parkinson’s disease and attention-deficit hyperactivity disorder. Cell Transplant. 29:963689720947416. 10.1177/0963689720947416 33028106 PMC7784516

[B29] Faraone S. V. Asherson P. Banaschewski T. Biederman J. Buitelaar J. K. Ramos-Quiroga J. A.. (2015). Attention-deficit/hyperactivity disorder. Nat. Rev. Dis. Primers 1:15020. 10.1038/nrdp.2015.20 27189265

[B30] Fayyad J. Sampson N. A. Hwang I. Adamowski T. Aguilar-Gaxiola S. Al-Hamzawi A.. (2017). The descriptive epidemiology of DSM-IV Adult ADHD in the World Health Organization World Mental Health Surveys. Atten. Defic. Hyperact. Disord. 9, 47–65. 10.1007/s12402-016-0208-3 27866355 PMC5325787

[B31] Feldman A. L. Johansson A. L. V. Gatz M. Flensburg M. Petzinger G. M. Widner H.. (2012). Accuracy and sensitivity of parkinsonian disorder diagnoses in two Swedish national health registers. Neuroepidemiology 38, 186–193. 10.1159/000336356 22472568 PMC3375128

[B32] Ferguson L. W. Rajput A. H. Rajput A. (2016). Early-onset vs. late-onset Parkinson’s disease: a clinical-pathological study. Can. J. Neurol. Sci. 43, 113–119. 10.1017/cjn.2015.244 26189779

[B33] Fluegge K. Fluegge K. (2018). Antecedent ADHD, dementia and metabolic dysregulation: A U.S. based cohort analysis. Neurochem. Int. 112, 255–258. 10.1016/j.neuint.2017.08.005 28811268

[B34] Fuemmeler B. F. Østbye T. Yang C. McClernon F. J. Kollins S. H. (2011). Association between attention-deficit/hyperactivity disorder symptoms and obesity and hypertension in early adulthood: a population-based study. Int. J. Obes. 35, 852–862. 10.1038/ijo.2010.214 20975727 PMC3391591

[B35] Galéra C. Salla J. Montagni I. Hanne-Poujade S. Salamon R. Grondin O.. (2017). Stress, attention deficit hyperactivity disorder (ADHD) symptoms and tobacco smoking: the i-share study. Eur. Psychiatry 45, 221–226. 10.1016/j.eurpsy.2017.07.007 28957791

[B36] Garcia-Argibay M. Pandya E. Ahnemark E. Werner-Kiechle T. Andersson L. M. Larsson H.. (2021). Healthcare utilization and costs of psychiatric and somatic comorbidities associated with newly diagnosed adult ADHD. Acta Psychiatr. Scand. 144, 50–59. 10.1111/acps.13297 33749845

[B37] Gibbins C. Weiss M. D. Goodman D. W. Hodgkins P. S. Landgraf J. M. Faraone S. V. (2010). ADHD-hyperactive/impulsive subtype in adults. Ment. Illn. 2, e9–e9. 10.4081/mi.2010.e9 25478092 PMC4253348

[B38] Ginsberg Y. Quintero J. Anand E. Casillas M. Upadhyaya H. P. (2014). Underdiagnosis of attention-deficit/hyperactivity disorder in adult patients: a review of the literature. Prim Care Companion CNS Disord. 16:PCC.13r01600. 10.4088/PCC.13r01600 25317367 PMC4195639

[B39] Golimstok A. Rojas J. I. Romano M. Zurru M. C. Doctorovich D. Cristiano E. (2011). Previous adult attention-deficit and hyperactivity disorder symptoms and risk of dementia with Lewy bodies: a case–control study. Eur. J. Neurol. 18, 78–84. 10.1111/j.1468-1331.2010.03064.x 20491888

[B40] Gomperts S. N. (2016). Lewy Body Dementias. Continuum 22, 435–463. 10.1212/CON.0000000000000309 27042903 PMC5390937

[B41] Goodman D. W. Mitchell S. Rhodewalt L. Surman C. B. H. (2016). Clinical presentation, diagnosis and treatment of attention-deficit hyperactivity disorder (ADHD) in older adults: a review of the evidence and its implications for clinical care. Drugs Aging 33, 27–36. 10.1007/s40266-015-0327-0 26659731

[B42] Goulardins J. B. Marques J. C. B. De Oliveira J. A. (2017). Attention deficit hyperactivity disorder and motor impairment. Percept. Mot. Skills 124, 425–440. 10.1177/0031512517690607 28361657

[B43] Haaxma C. A. Bloem B. R. Borm G. F. Oyen W. J. G. Leenders K. L. Eshuis S.. (2007). Gender differences in Parkinson’s disease. J. Neurol. Neurosurg. Psychiatry 78, 819–824. 10.1136/jnnp.2006.103788 17098842 PMC2117736

[B44] Harding Z. Wilkinson T. Stevenson A. Horrocks S. Ly A. Schnier C.. (2019). Identifying Parkinson’s disease and parkinsonism cases using routinely collected healthcare data: a systematic review. PLoS One 14:e0198736. 10.1371/journal.pone.0198736 30703084 PMC6354966

[B45] Hartsough C. S. Babinski L. M. Lambert N. M. (1996). Tracking procedures and attrition containment in a long-term follow-up of a community-based ADHD sample. J. Child Psychol. Psychiatry 37, 705–713. 10.1111/j.1469-7610.1996.tb01462.x 8894951

[B46] Hjerpe P. Merlo J. Ohlsson H. Bengtsson Boström K. Lindblad U. (2010). Validity of registration of ICD codes and prescriptions in a research database in Swedish primary care: a cross-sectional study in Skaraborg primary care database. BMC Med. Inform. Decis. Mak. 10:23. 10.1186/1472-6947-10-23 20416069 PMC2864192

[B47] Irmisch G. Richter J. Thome J. Sheldrick A. J. Wandschneider R. (2013). Altered serum mono- and polyunsaturated fatty acid levels in adults with ADHD. ADHD Atten. Deficit Hyperact. Disord. 5, 303–311. 10.1007/s12402-013-0107-9 23564274

[B48] Ismail Z. Gatchel J. Bateman D. R. Barcelos-Ferreira R. Chantillon M. Jaeger J.. (2018). Affective and emotional dysregulation as pre-dementia risk markers: exploring the mild behavioral impairment symptoms of depression, anxiety, irritability and euphoria. Int. Psychogeriatrics 30, 185–196. 10.1017/S1041610217001880 28899446

[B49] Ivanchak N. Fletcher K. Jicha G. (2012). Attention-deficit/hyperactivity disorder in older adults: prevalence and possible connections to mild cognitive impairment. Curr. Psychiatry Rep. 14, 552–560. 10.1007/s11920-012-0305-8 22886581 PMC3718885

[B50] Jetté N. Quan H. Hemmelgarn B. Drosler S. Maass C. Moskal L.. (2010). The development, evolution and modifications of ICD-10: challenges to the international comparability of morbidity data. Med. Care 48, 1105–1110. 10.1097/MLR.0b013e3181ef9d3e 20978452

[B51] Kieling R. Rohde L. A. (2012). ADHD in children and adults: diagnosis and prognosis. Curr. Top. Behav. Neurosci. 9, 1–16. 10.1007/7854_2010_115 21499858

[B52] Kompliti K. Goetz C. G. (1998). Hyperkinetic movement disorders misdiagnosed as tics in gilles de la tourette syndrome. Mov. Disord. 13, 477–480. 10.1002/mds.870130317 9613740

[B53] Konofal E. Lecendreux M. Cortese S. (2010). Sleep and ADHD. Sleep Med. 11, 652–658. 10.1016/j.sleep.2010.02.012 20620109

[B54] Kooij J. J. S. Michielsen M. Kruithof H. Bijlenga D. (2016). ADHD in old age: a review of the literature and proposal for assessment and treatment. Expert Rev. Neurother. 16, 1371–1381. 10.1080/14737175.2016.1204914 27334252

[B55] Lee Y.-K. Hou S.-W. Lee C.-C. Hsu C.-Y. Huang Y.-S. Su Y.-C. (2013). Increased risk of dementia in patients with mild traumatic brain injury: a nationwide cohort study. PLoS One 8:e62422. 10.1371/journal.pone.0062422 23658727 PMC3641064

[B56] Lindemann C. Langner I. Banaschewski T. Garbe E. Mikolajczyk R. T. (2017). The risk of hospitalizations with injury diagnoses in a matched cohort of children and adolescents with and without attention deficit/hyperactivity disorder in Germany: a database study. Front. Pediatr. 5:220. 10.3389/fped.2017.00220 29114538 PMC5660679

[B57] Liou Y.-J. Wei H.-T. Chen M.-H. Hsu J.-W. Huang K.-L. Bai Y.-M.. (2018). Risk of traumatic brain injury among children, adolescents and young adults with attention-deficit hyperactivity disorder in taiwan. J. Adolesc. Heal. 63, 233–238. 10.1016/j.jadohealth.2018.02.012 29970331

[B58] Livingston G. Huntley J. Sommerlad A. Ames D. Ballard C. Banerjee S.. (2020). Dementia prevention, intervention and care: 2020 report of the lancet commission. Lancet 396, 413–446. 10.1016/S0140-6736(20)30367-6 32738937 PMC7392084

[B59] May T. Adesina I. McGillivray J. Rinehart N. J. (2019). Sex differences in neurodevelopmental disorders. Curr. Opin. Neurol. 32, 622–626. 10.1097/WCO.0000000000000714 31135460

[B60] Mazzali C. Paganoni A. M. Ieva F. Masella C. Maistrello M. Agostoni O.. (2016). Methodological issues on the use of administrative data in healthcare research: the case of heart failure hospitalizations in Lombardy region, 2000 to 2012. BMC Health. Serv. Res. 16:234. 10.1186/s12913-016-1489-0 27391599 PMC4939041

[B61] McKeith I. G. Boeve B. F. Dickson D. W. Halliday G. Taylor J.-P. Weintraub D.. (2017). Diagnosis and management of dementia with Lewy bodies: fourth consensus report of the DLB Consortium. Neurology 89, 88–100. 10.1212/WNL.0000000000004058 28592453 PMC5496518

[B62] McKeith I. G. Galasko D. Kosaka K. Perry E. K. Dickson D. W. Hansen L. A.. (1996). Consensus guidelines for the clinical and pathologic diagnosis of dementia with Lewy bodies (DLB): report of the consortium on DLB international workshop. Neurology 47, 1113–1124. 10.1212/wnl.47.5.1113 8909416

[B64] McKhann G. Drachman D. Folstein M. Katzman R. Price D. Stadlan E. M. (1984). Clinical diagnosis of Alzheimer’s disease: report of the NINCDS-ADRDA work group* under the auspices of department of health and human services task force on Alzheimer’s disease. Neurology 34, 939–939. 10.1212/wnl.34.7.939 6610841

[B63] McKhann G. M. Knopman D. S. Chertkow H. Hyman B. T. Jack C. R. Kawas C. H.. (2011). The diagnosis of dementia due to Alzheimer’s disease: recommendations from the national institute on aging-Alzheimer’s association workgroups on diagnostic guidelines for Alzheimer’s disease. Alzheimers Dement. 7, 263–269. 10.1016/j.jalz.2011.03.005 21514250 PMC3312024

[B65] Mehanna R. Moore S. Hou J. G. Sarwar A. I. Lai E. C. (2014). Comparing clinical features of young onset, middle onset and late onset Parkinson’s disease. Parkinsonism. Relat. Disord. 20, 530–534. 10.1016/j.parkreldis.2014.02.013 24631501

[B66] Michielsen M. Semeijn E. Comijs H. C. Van De Ven P. Beekman A. T. F. Deeg D. J. H.. (2012). Prevalence of attention-deficit hyperactivity disorder in older adults in the netherlands. Br. J. Psychiatry 201, 298–305. 10.1192/bjp.bp.111.101196 22878132

[B67] Mouton A. Blanc F. Gros A. Manera V. Fabre R. Sauleau E.. (2018). Sex ratio in dementia with Lewy bodies balanced between Alzheimer’s disease and Parkinson’s disease dementia: a cross-sectional study. Alzheimers Res. Ther. 10:92. 10.1186/s13195-018-0417-4 30208961 PMC6136211

[B68] Muzerengi S. Rick C. Begaj I. Ives N. Evison F. Woolley R. L.. (2017). Coding accuracy for Parkinson’s disease hospital admissions: implications for healthcare planning in the UK. Public Health 146, 4–9. 10.1016/j.puhe.2016.12.024 28404473

[B69] Newcorn J. H. Weiss M. Stein M. A. (2007). The complexity of ADHD: diagnosis and treatment of the adult patient with comorbidities. CNS Spectr. 12, 1–6. 10.1017/s1092852900026158 17667893

[B70] O’Malley K. J. Cook K. F. Price M. D. Wildes K. R. Hurdle J. F. Ashton C. M. (2005). Measuring diagnoses: ICD code accuracy. Health Serv. Res. 40, 1620–1639. 10.1111/j.1475-6773.2005.00444.x 16178999 PMC1361216

[B71] Otero Varela L. Doktorchik C. Wiebe N. Quan H. Eastwood C. (2021). Exploring the differences in ICD and hospital morbidity data collection features across countries: an international survey. BMC Health. Serv. Res. 21:308. 10.1186/s12913-021-06302-w 33827567 PMC8025494

[B72] Polanczyk G. de Lima M. S. Horta B. L. Biederman J. Rohde L. A. (2007). The worldwide prevalence of ADHD: a systematic review and metaregression analysis. Am. J. Psychiatry 164, 942–948. 10.1176/ajp.2007.164.6.942 17541055

[B73] Pollack J. (2012). Distinguishing between adult ADHD and mild cognitive impairment. Curr. Psychiatry 8, 48–48.

[B74] Post B. van den Heuvel L. van Prooije T. van Ruissen X. van de Warrenburg B. Nonnekes J. (2020). Young onset Parkinson’s disease: a modern and tailored approach. J. Parkinsons Dis. 10, S29–S36. 10.3233/JPD-202135 32651336 PMC7592661

[B75] Ramsay J. R. (2017). Assessment and monitoring of treatment response in adult ADHD patients: current perspectives. Neuropsychiatr. Dis. Treat. 13, 221–232. 10.2147/NDT.S104706 28184164 PMC5291336

[B76] Retz-Junginger P. Retz W. Blocher D. Weijers H.-G. Trott G.-E. Wender P. H.. (2002). Wender utah rating scale (WURS-k) die deutsche kurzform zur retrospektiven erfassung des hyperkinetischen syndroms bei erwachsenen. Nervenarzt 73, 830–838. 10.1007/s00115-001-1215-x 12215873

[B77] Rosler M. Casas M. Konofal E. Buitelaar J. (2010). Attention deficit hyperactivity disorder in adults. World J. Biol. Psychiatry 11, 684–698. 10.3109/15622975.2010.483249 20521876

[B78] Salvi V. Migliarese G. Venturi V. Rossi F. Torriero S. Viganò V.. (2019). ADHD in adults: clinical subtypes and associated characteristics. Riv. Psichiatr. 54, 84–89. 10.1708/3142.31249 30985833

[B79] Schrag A. Schott J. M. (2006). Epidemiological, clinical and genetic characteristics of early-onset parkinsonism. Lancet Neurol. 5, 355–363. 10.1016/S1474-4422(06)70411-2 16545752

[B80] Sezgin M. Bilgic B. Tinaz S. Emre M. (2019). Parkinson’s disease dementia and lewy body disease. Semin. Neurol. 39, 274–282. 10.1055/s-0039-1678579 30925619

[B81] Sharma M. J. Lavoie S. Callahan B. L. (2021). A call for research on the validity of the age-of-onset criterion application in older adults being evaluated for ADHD: a review of the literature in clinical and cognitive psychology. Am. J. Geriatr. Psychiatry 29, 669–678. 10.1016/j.jagp.2020.10.016 33191098

[B82] Sharp E. S. Gatz M. (2011). Relationship between education and dementia: an updated systematic review. Alzheimer Dis. Assoc. Disord. 25, 289–304. 10.1097/WAD.0b013e318211c83c 21750453 PMC3193875

[B83] Shin H.-W. Chung S. J. (2012). Drug-induced parkinsonism. J. Clin. Neurol. 8, 15–21. 10.3988/jcn.2012.8.1.15 22523509 PMC3325428

[B84] Sibley M. H. (2021). Empirically-informed guidelines for first-time adult ADHD diagnosis. J. Clin. Exp. Neuropsychol. 43, 340–351. 10.1080/13803395.2021.1923665 33949916

[B85] Sibley M. H. Pelham W. E. Molina B. S. G. Gnagy E. M. Waxmonsky J. G. Waschbusch D. A.. (2012). When diagnosing ADHD in young adults emphasize informant reports, DSM items and impairment. J. Consult. Clin. Psychol. 80, 1052–1061. 10.1037/a0029098 22774792 PMC3919146

[B86] Sibley M. H. Rohde L. A. Swanson J. M. Hechtman L. T. Molina B. S. G. Mitchell J. T.. (2017). Late-onset ADHD reconsidered with comprehensive repeated assessments between ages 10 and 25. Am. J. Psychiatry 175, 140–149. 10.1176/appi.ajp.2017.17030298 29050505 PMC5814300

[B87] St. Germaine-Smith C. Metcalfe A. Pringsheim T. Roberts J. I. Beck C. A. Hemmelgarn B. R.. (2012). Recommendations for optimal ICD codes to study neurologic conditions. Neurology 79, 1049–1055. 10.1212/WNL.0b013e3182684707 22914826 PMC3430709

[B88] Swarztrauber K. Anau J. Peters D. (2005). Identifying and distinguishing cases of parkinsonism and Parkinson’s disease using ICD-9 CM codes and pharmacy data. Mov. Disord. 20, 964–970. 10.1002/mds.20479 15834854

[B89] Tani P. Lindberg N. Appelberg B. Nieminen-von Wendt T. von Wendt L. Porkka-Heiskanen T. (2006). Childhood inattention and hyperactivity symptoms self-reported by adults with asperger syndrome. Psychopathology 39, 49–54. 10.1159/000089910 16299413

[B90] Thorell L. B. Holst Y. Chistiansen H. Kooij J. J. S. Bijlenga D. Sjöwall D. (2017). Neuropsychological deficits in adults age 60 and above with attention deficit hyperactivity disorder. Eur. Psychiatry 45, 90–96. 10.1016/j.eurpsy.2017.06.005 28750278

[B91] Tzeng N.-S. Chung C.-H. Lin F.-H. Yeh C.-B. Huang S.-Y. Lu R.-B.. (2019). Risk of dementia in adults with ADHD: a nationwide, population-based cohort study in Taiwan. J. Atten. Disord. 23, 995–1006. 10.1177/1087054717714057 28629260

[B92] Vassilaki M. Aakre J. A. Cha R. H. Kremers W. K. St. Sauver J. L. Mielke M. M.. (2015). Multimorbidity and risk of mild cognitive impairment. J. Am. Geriatr. Soc. 63, 1783–1790. 10.1111/jgs.13612 26311270 PMC4607039

[B93] Volkow N. D. Swanson J. M. (2013). Adult attention deficit-hyperactivity disorder. N Engl. J. Med. 369, 1935–1944. 10.1056/NEJMcp1212625 24224626 PMC4827421

[B94] Walitza S. Melfsen S. Herhaus G. Scheuerpflug P. Warnke A. Muller T.. (2007). Association of Parkinson’s disease with symptoms of attention deficit hyperactivity disorder in childhood. J. Neural Transm. 72, 311–315. 10.1007/978-3-211-73574-9_38 17982908

[B95] Wells G. A. Shea B. O’Connell D. T. Peterson J. Welch V. Losos M.. (2011). The Newcastle-Ottawa Scale (NOS) for Assessing the Quality of Nonrandomised Studies in Meta-Analyses. Ottawa, ON: The Ottawa Hospital. Available online at: http://www.ohri.ca/programs/clinical_epidemiology/oxford.asp.

[B96] Wermuth L. Lassen C. F. Himmerslev L. Olsen J. Ritz B. (2012). Validation of hospital register-based diagnosis of Parkinson’s disease. Dan. Med. J. 59:A4391. 22381086 PMC3643969

[B97] Wichmann T. DeLong M. R.. (2010). “Basal ganglia, functional organization,” in *Encyclopedia of Movement Disorders*, eds Kompoliti K. Metman L. V.. Oxford: Academic Press, 118–125. Available online at: https://www.sciencedirect.com/science/article/pii/B9780123741059004378.

[B98] Wickremaratchi M. M. Perera D. O’Loghlen C. Sastry D. Morgan E. Jones A.. (2009). Prevalence and age of onset of Parkinson’s disease in Cardiff: a community based cross sectional study and meta-analysis. J. Neurol. Neurosurg. Psychiatry 80, 805–807. 10.1136/jnnp.2008.162222 19531689

[B99] Wilkinson T. Ly A. Schnier C. Rannikmäe K. Bush K. Brayne C.. (2018). Identifying dementia cases with routinely collected health data: A systematic review. Alzheimer’s Dement. 14, 1038–1051. 10.1016/j.jalz.2018.02.016 29621480 PMC6105076

[B100] Ylönen S. Siitonen A. Nalls M. A. Ylikotila P. Autere J. Eerola-Rautio J.. (2017). Genetic risk factors in Finnish patients with Parkinson’s disease. Parkinsonism. Relat. Disord. 45, 39–43. 10.1016/j.parkreldis.2017.09.021 29029963 PMC5812481

[B101] Zhang L. Du Rietz E. Kuja-Halkola R. Dobrosavljevic M. Johnell K. Pedersen N. L.. (2021). Attention-deficit/hyperactivity disorder and Alzheimer’s disease and any dementia: a multi-generation cohort study in Sweden. Alzheimer’s Dement. [Online ahead of print]. 10.1002/alz.12462 34498801

[B102] Zucker M. Morris M. K. Ingram S. M. Morris R. D. Bakeman R. (2002). Concordance of self- and informant ratings of adults’ current and childhood attention-deficit/hyperactivity disorder symptoms. Psychol. Assess. 14, 379–389. 10.1037//1040-3590.14.4.379 12501563

